# Integrated Methodology from Synthesis to *in Vivo* Study that Identifies Nanostructure Shape “Hot Spots” in T Cell Receptor Repertoire

**DOI:** 10.1021/acs.nanolett.5c00741

**Published:** 2025-04-21

**Authors:** Yanqiu Ye, Guohui Huang, Wei Zhang, Jiasheng Wu, Jianhao Wu, Yingxin Li, Xiaoxia Zhou, Jianbo Jia, Zengchun Xie, Bing Yan, Kenneth A. Dawson, Jingqi Chen, Yi-Feng Wang, Yan Yan

**Affiliations:** † Guangzhou Key Laboratory for Research and Development of Nano-Biomedical Technology for Diagnosis and Therapy, Guangdong Provincial Education Department Key Laboratory of Nano-Immunoregulation Tumor Microenvironment, Department of Oncology & Translational Medicine Center, The Second Affiliated Hospital & the Affiliated Cancer Hospital, Guangzhou Medical University, Guangzhou, Guangdong 510260, P.R. China; ∇ Guangzhou Key Laboratory for Research and Development of Nano-Biomedical Technology for Diagnosis and Therapy, Guangdong Provincial Education Department Key Laboratory of Nano-Immunoregulation Tumor Microenvironment, Department of Oncology & Translational Medicine Center, The Second Affiliated Hospital, Guangzhou Medical University, Guangzhou, Guangdong 510260, P.R. China; ‡ Centre for BioNano Interactions, School of Chemistry, University College Dublin, Belfield, Dublin 4, Ireland; § School of Biomolecular and Biomedical Science, UCD Conway Institute of Biomolecular and Biomedical Research, University College Dublin, Belfield, Dublin 4, Ireland; ∥ Institute of Environmental Research at Greater Bay Area, Key Laboratory for Water Quality and Conservation of the Pearl River Delta, Ministry of Education, Guangzhou University, Guangzhou 510006, P.R. China

**Keywords:** Nanoscale shape, Nanoscale biological recognition, T cell receptor repertoire, Clonal diversity, Vaccine adjuvants

## Abstract

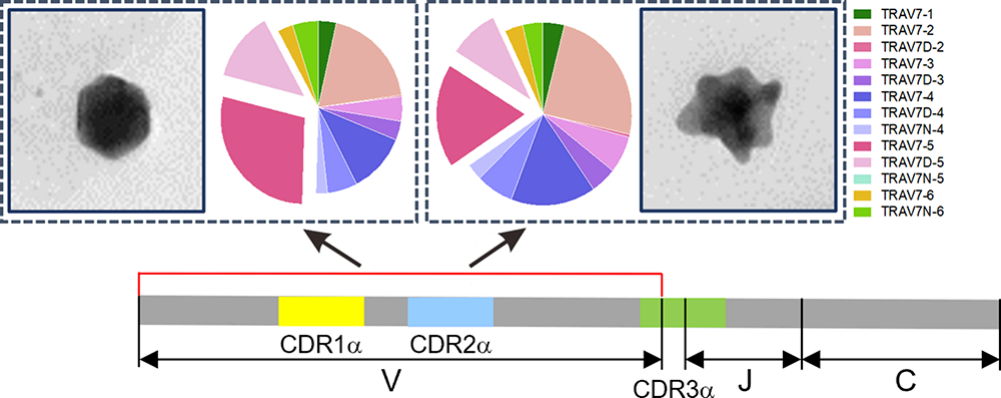

A new integrated tunable microfluidic particle synthesis and shape population analysis workflow allows us to study the immunological readouts for even highly complex shaped nanoparticles. Using this approach, we demonstrate that some gold nanoparticles, when injected parenterally, are taken up by axillary and brachial lymph nodes. We then show that specific nanoparticle shapes influence the primary structure of the T cell receptor, inducing changes in hypervariable complementary-determining regions (CDRs) and increasing the clonal diversity of the T cell receptor repertoires. These same particles were previously found to modify cellular epigenomes and elevate the level of autoantibodies. Our results are consistent with other emerging reports that precisely controlled nanoarchitectural features are recognized and captured in multiple tiers of biology, with potential implications for vaccine adjuvant design. Our conclusions may also be relevant to an extensive legacy of poorly understood epidemiological studies, suggesting links between some pollutant particulates and complex forms of immune dysregulation and autoimmune diseases.

Recent investigations suggest the presence of novel mechanisms that allow living organisms to “recognize” (capture and retain) extended information about nanoscale architecture defined across some hundreds of nanometers.^1^ The observation that (in contrast to molecular recognition) precise control of structural features on such extended scales can induce profound and multitiered biological responses appears to be of fundamental significance.^2−7^ It is also noteworthy that (in cell studies) these effects appear partially independent of the biological milieu (and thereby biomolecular surface corona presentation),^8^ suggesting some degree of decoupling or semi-independent roles for the shape-architecture and biomolecular surface recognition.^1,6^ Both cell and *in vivo* studies suggest far-reaching immunological consequences of this form of recognition, pointing toward practical implications and novel applications of these degrees of control.

Recognizing the challenges in drawing reproducible causal connections between complex particle architectures and biological outcomes, we have sought to draw these observations into a quantitative and reproducible methodological framework that combines tunable microfluidic nanoparticle synthesis, nanoscale architecture analysis, and high-throughput biological readouts. These are then connected into an inductive search loop to identify specific shape regimes that induce profound transcriptome changes in antigen presenting cells (APCs).^5,6^ It is important to stress that this enables a level of reproducibility and transferability of results across different studies that are rarely achieved for the population of complex particle architectures in biology. This makes it possible to identify unique shape populations among an evolving family of related shape distributions that exhibit distinctive biological behaviors. These developments in the synthesis, capture of particle architectures, and screening of biological read-outs have made it possible to identify specific architectures that cause distinctive transcriptome regulation, induce highly specific histone modifications in innate immune cells,^7^ and *in vivo* lead to new pools of antibodies.^6^ Here, we show examples in which such shaped “hot spots” lead to distinctive T cell receptor (TCR) repertoires. Taken together, these observations suggest a mechanistic connection between nanoscale shape and trained immunity.

The full implications of these emerging observations will only emerge over time, but it should be noted that particles (likely via their nanoscale interactions) have long played a role as vaccine adjuvants.^9^ While some of the approved adjuvant materials have been successfully used for many years, their real role remains unclear. For instance, the action of alum as an adjuvant has been explained by various mechanisms, including activation of the kinase cascade,^10^ inflammasome activation,^11^ cell death, and DNA release.^12^ Furthermore, several physical parameters of particle adjuvants, such as particle shape, surface chemistry, and crystallinity,^13−15^ are believed to influence the activation of innate sensing pathways, suggesting the immune system can be stimulated by mechanisms beyond the known innate sensing pathways (e.g., pattern recognition receptors). It seems most likely that multiple mechanisms are involved, perhaps explaining the difficulty in coming to a simple explanation of their success. Although most adjuvants are known to work primarily by stimulating innate immune cells to induce cytokine secretion and antigen presentation, growing evidence points to a role in direct activation of adaptive immune cells.^9,16−18^ However, information about the effects of particle adjuvants on T cells is very sparse. Indeed, while we have a partial understanding of shape recognition in APCs, at present, we do not know if (let alone how) spatially extended interactions (on the scale of some tens of nanometers) are relayed and reflected in T cell populations. This is a rather fundamental question of principle, so we decided to plan broad investigations with a proof-of-concept study that could capture suggestive readouts. We believe that the outcomes are sufficiently striking and present them here.

We undertook a systemic exploration of the TCR repertoire as a likely footprint of the complex direct or indirect roles of particle architecture in adaptive immunity. TCRs are heterodimers composed of α- and β-chains expressed by most T cells or γ- and δ-chains expressed by a small proportion of T cells at mucosal sites.^19^ Therefore, our study investigated the distribution and clonality of variable regions of TCR α- and β-chains, which are assembled by somatic recombination from variable (V), diversity (D, only for β-chain), and joining (J) segments. Using the microfluidic synthesis platform, we are able to fine-tune a set of distinct gold nanoparticle shape ensembles (GNPs) stretching across a shape regime that was identified as immunologically interesting in our previous studies. We now show that in mice the same nanoscale shape regime that was shown to activate innate immune cells also leads to significant changes in gene usage of V segments of both α- and β-chains, as well as an elevation of the diversity of the TCR repertoire. This suggests that specific shape-dependent regulations can be very efficiently captured and relayed via the TCR repertoires. Recognition of highly specific spatially extended features of particle architecture may play a much broader immunological role than has so far been supposed.

## Experimental Setup for TCR Repertoire Analysis

**Figure 1:**
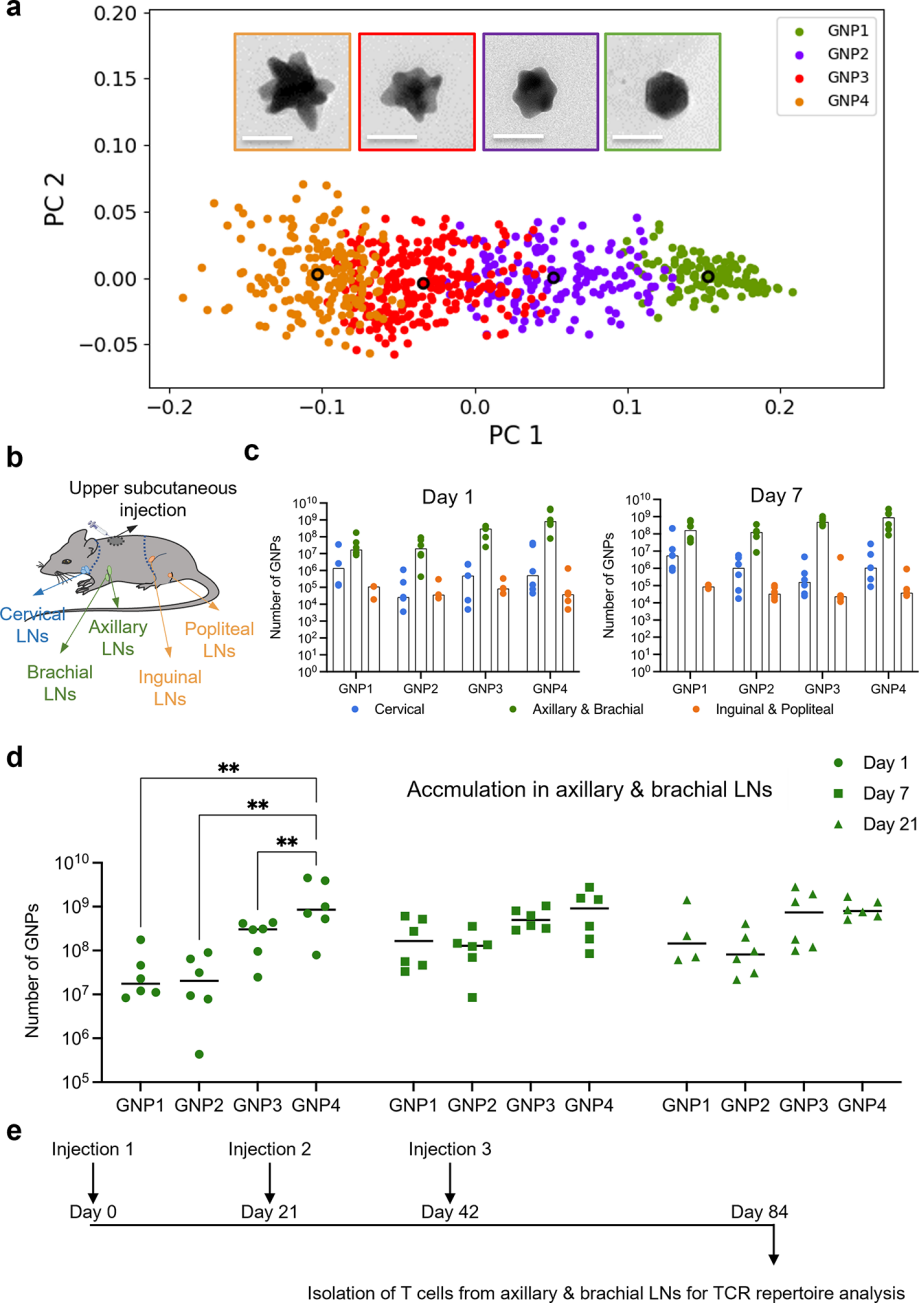
Experimental setup for TCR repertoire analysis. (a) Shape contour analysis of GNPs. Representative TEM images showing the center of gravity of each shape ensemble. Scale bar, 50 nm. (b) Schematic illustration of injection site and three groups of relevant draining LNs shown in different colors. (c) Accumulation of injected GNPs in the draining LNs measured by ICP-MS, confirming axillary and brachial LNs are the major draining LNs. Each point represents an individual animal. Bars represent the median. (d) Accumulation of GNPs in axillary and brachial LNs over extended time periods. Each point represents an individual animal. Lines represent the median. Ordinary two-way ANOVA with Tukey’s multiple comparisons test. **Adjusted *p*-value < 0.01. (e) Immunization schedule.

Conventional batch syntheses of complex particulates are rarely sufficiently controllable or reproducible for the present purpose, so here, we used tunable microfluidic synthetic control devices (Figure S1a,b) allied to the quantitative characterization of nanoscale shape ensembles (populations).^5,6^ The synthetic approach has been previously described in detail in refs (5 and 6), and we briefly summarize key elements here. In refs (5 and 6), we described how the evolution of microfluidic parameters allowed the search of a larger homologous series of “spiky” structures to identify those of most immunological interest, and those (now renamed GNP1–4; see Figure 1a) are now the subject of the present article. First, the production of highly reproducible five nanometer gold seeds (Figure S1c,d, reproduced from ref (6)) is found to be a key step in the successful growth of downstream nanostructures (Figure S1e,f). Population-level characterization (described in ref (6), first introduced in ref (5)) allowed us to precisely recapitulate specific shape regimes and carry them over between different types of investigation, assigning different biological outcomes to particle populations, including those that visually appear to be quite similar.^5^ For the current work, we synthesized seeds that were indistinguishable from those in previous publications (Figure S1g). Then, another microfluidic reactor (Figure S1b) used identical conditions to those in ref (6) to prepare the shapes studied here. Figure S1h illustrates how the identified immunological “hot spot” structure identified in previous screening studies (in ref (6) named MR_GNP07, now named GNP3) is reproduced. Since previous studies have identified some forms of T cell activation in the presence of nonfunctionalized ultrasmall silica nanoparticles,^20,21^ we noted that residual seeds were absent in GNP3 (and all other) shaped GNPs (Figure S1g). This interesting observation further illustrates the importance of small volume mixing of seeds and reagents in suppressing growth fluctuation and driving seed growth to completion. It is also worth noting that considerable efforts were made throughout the whole study to eliminate spurious causes of distinctive biological read-outs including ultrasmall seed contamination (Figure S1h), particle aggregation,^22^ and also residual reactants adsorbed to the surface. Aggregation was excluded (Table S1) at least up to and at the point of injection. While it is not possible to entirely exclude the presence of vanishingly small surface residuals, usual sensitive chemicals and surface analyses excluded the presence of detectable levels of known forms of contamination.

Then, by tuning the microfluidic synthesis conditions, we generated the sequence of the GNP (GNP1–GNP4, Figure 1a) ensemble trajectory that traverses a key shape regime (GNP3) that was previously shown to induce significant transcriptome and epigenomic changes in APCs in vitro, making that an interesting candidate for T cell studies. In Figure 1a, the reader may consult previous reports (e.g., refs (5 and 6)) for the detailed concept of principal components of the Fourier transform coefficients of the (projected) shapes. However, (heuristically) for the present purpose, while PC1 and PC2 mainly capture simpler features (e.g., projected size and basic shape) of particles, details of the tips and distances between them are captured in higher principal components. As features that drive distinct biological effects are strongly associated with tips and distances between them, particle populations differentiated by PC1 and PC2 do not necessarily present the categories of biological responses. To simply illustrate the meaning of these shapes (and quantitative shape ensemble progression as a trajectory), representative transmission electron microscopy (TEM) images of the “center of gravity” of the shape ensemble are also shown in Figure 1a. It is crucial that all GNP1–GNP4s were made in a laminar flow hood and endotoxin-free, which is of significant importance in preventing immune activation caused by endotoxin contamination (Figure S2).

We then sought to investigate the lymphatic accumulation of those particle ensembles following subcutaneous injection into healthy mice (Figure 1b). We used well characterized particles from the same population as the rest of the experiments reported in this paper. We then harvested all visible peripheral lymph nodes (LNs) in the head and neck region (i.e., cervical LNs), at the forelimb (i.e., axillary and brachial LNs), and at the hindlimb (i.e., inguinal and popliteal LNs). GNP accumulation was quantified by Inductively Coupled Plasma-Mass Spectrometry (ICP-MS). We observed that the axillary and brachial LNs were the major draining LNs, with 10–100 times higher GNP levels than in the cervical LNs and 10^3^–10^4^ times higher than in the popliteal and inguinal LNs (Figure 1c). Furthermore, GNP4 exhibited higher accumulation in the axillary and brachial LNs on Day 1 in comparison with less branched GNP1, GNP2, and GNP3. On Day 7 and 21, all four shape ensembles had a comparable level of lymphatic accumulation (Figure 1d). This could suggest that the more highly branched shape is associated with faster drainage, although we would require more quantitative information on uptake to establish that point.

Based on these preliminary investigations, we subcutaneously immunized the mice with GNPs, followed by two boosts spaced every 3 weeks (Figure 1e). The axillary and brachial LNs were collected 6 weeks after the second boost. To avoid incomplete recovery of T cells, negative isolation was used to enrich the T cells, and the resulting T cell purity was confirmed by immunostaining with pan T cell marker CD3e (Figure S3).

## TCR V Distribution Influenced by the Nanoscale Shape GNP3

Total mRNA in the isolated T cells was extracted and reverse transcribed to cDNA. The Bioanalyzer 2100 analysis reveals that the total mRNA and the reverse transcribed cDNA remain intact, with no evidence of significant degradation (Figure S4). SMART technology (5′RACE) was used to fully capture and amplify variable regions of TCR α- and TCR β-cDNA and attach unique molecular identifiers for next-generation sequencing. Results of the reconstruction of V, D (only for β-chain), and J segments using two open-source algorithms, MiXCR^23^ and TRUST4,^24^ are consistent with each other.

**Figure 2:**
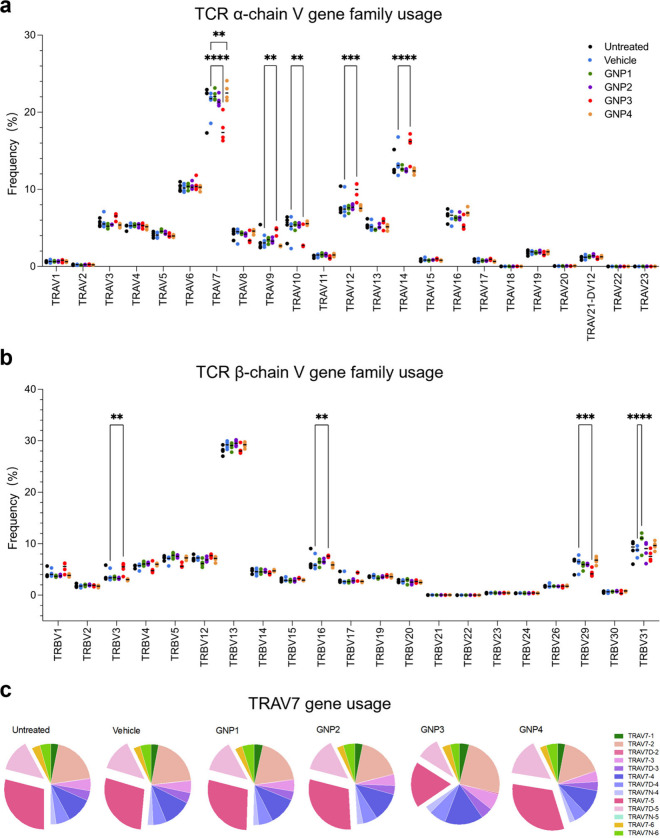
TCR V distribution influenced by the nanoscale shape of GNP3. (a, b) TRAV and TRBV gene usage. Each point represents an individual animal (*n* = 4, 2 female and 2 male). The line represents the median. *p*-values are calculated by two-way ANOVA. *p < 0.05; **p < 0.01; ***p < 0.005; ****p < 0.001. (c) Pie graphs depicting the average TRAV7 gene usage of the four mice.

Our conclusions are as follows. First, gene family usage of TRAJ, TRBD, and TRBJ segments did not exhibit a significant change between GNP-treatment groups and the vehicle (BSA saline). In contrast, GNP3 treatment induced significant shifts in distribution of five gene families in TRAV segments (Figure 2a) and three gene families in TRBV segments (Figure 2b). These include the most frequently used gene family (TRAV7) in the TRAV segment (over 20%). Specifically, gene usage of TRAV7-5 and TRAV7D-5 was significantly decreased (Figure 2c), leading to the overall decrease in the TRAV7 distribution.

## Length Distribution of CDR1α and CDR2α Altered by GNP3

**Figure 3:**
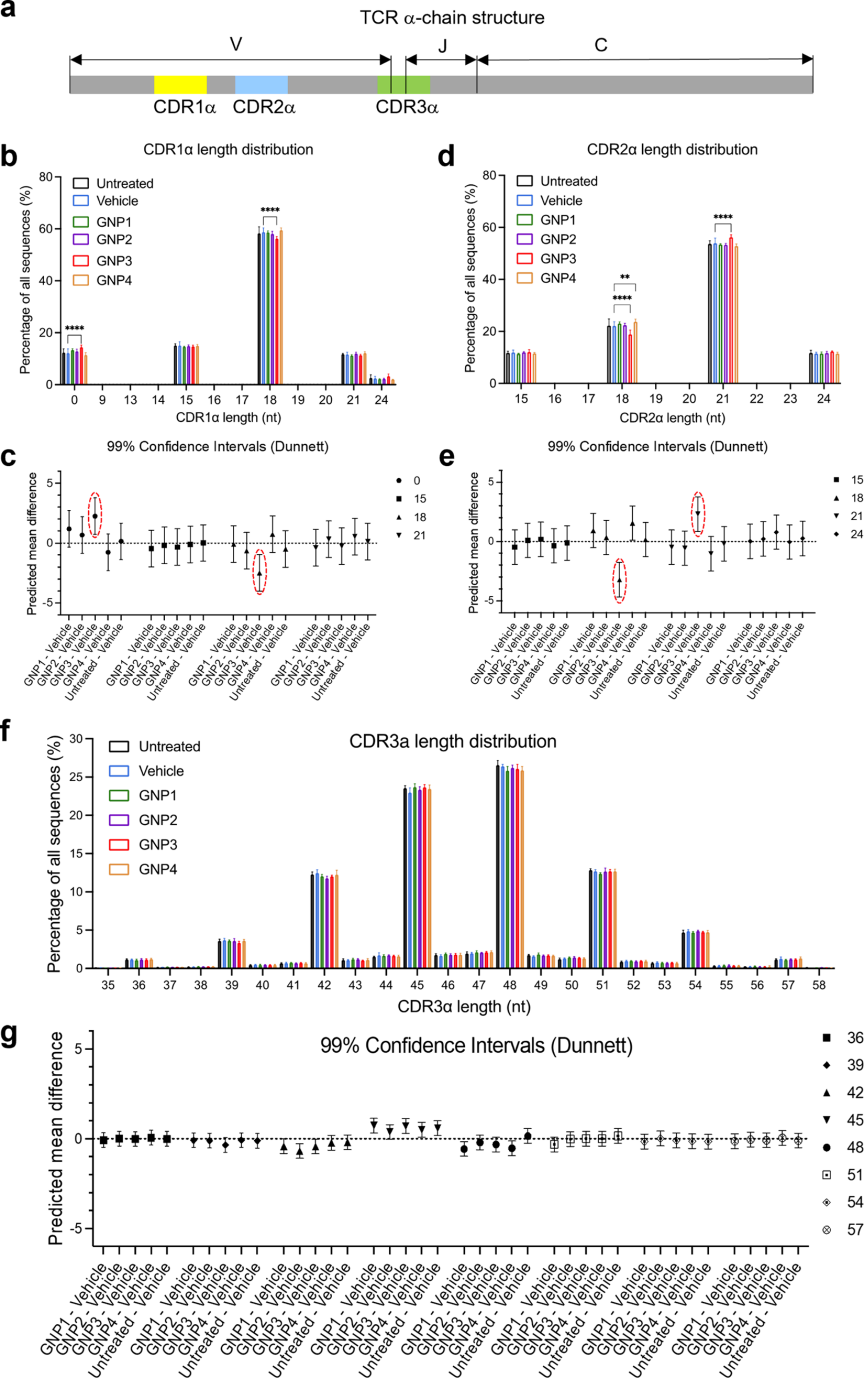
GNP3 induces shifts in length distribution of CDR1 and CDR2 of the TCR α-chain. (a) Scheme of the TCR α-chain structure to illustrate the germline V segment encoded-CDR1α and CDR2α. (b, d, and f) CDR length distribution. The data are expressed as mean standard deviation. (c, e, and g) Two-way ANOVA Dunnett’s multiple comparisons test to illustrate confidence intervals compared with the vehicle group. The most significant shifts are circled.

TCRs typically recognize short peptide antigens docked in major histocompatibility complexes (MHCs) to form the peptide-MHC complex (pMHC). Six short hairpin loops called complementarity-determining regions (CDRs) from the α- and β-chain account for the main interactions within the pMHC. CDR1 and CDR2 are encoded in the germline by the V segments, whereas CDR3s are generated by recombination of V, (D), and J segments (Figure 3a, showing CDRs in the TCR α-chain).^25^ TRUST4 is considered an effective method to infer full-length V(D)J sequences, so we employed that approach to analyze the six CDR sequences. While the length distribution of the three CDRs in the β-chain remains unchanged, significant shifts in CDR1α and CDR2α length distribution induced by GNP3 were detected (Figure 3b–e). For CDR1α, a decrease in the frequency at 6 amino acid lengths and an increase in the frequency at 0 amino acid length were observed (Figure 3c). For CDR2α, an increase of the frequency at 7 amino acid lengths and a decrease of the frequency at 6 amino acid lengths are observed (Figure 3e). In contrast, the length distribution of CDR3α only exhibited subtle changes (Figures 3f,g) and can be sensitively influenced by minor factors (such as, BSA saline as the vehicle group induced a small increase of the frequency at 15 amino acid length; see Figure 3g).

While changes in CDR3 are most often discussed in the context of peptide recognition,^26^ within the canonical TCR-pMHC docking model, it is believed that the germline encoded CDR1 and CDR2 domains are predominantly involved in the interaction with the MHC molecule itself. Growing evidence suggests that CDR1 and CDR2 play a somewhat more complex role in the formation of TCR-pMHC complexes. Indeed the reverse docking model implies that modification of recognition contacts (for example, additional spatial constraints on the peptides) can drive changes in specific V gene usage.^27−29^ The length distribution shifts we observed, along with increased TRAV14 and decreased TRAV7 gene usage, are consistent with that picture because TRAV7 codes CDR2α with 6 amino acids and TRAV14 codes CDR2α with 7 amino acids.^30^ While the detailed mechanisms still have to be unraveled, it will be important to investigate if the implied modified CDR1α- pMHC and CDR2α- pMHC interactions derive from physical constraints imposed by particle shape during antigen recognition.^31^ This could suggest a mechanism via which more extended information about the original nanostructure architecture is transferred into the pool of T cells.

## GNP3 Increases TCR Clonal Diversity

**Figure 4:**
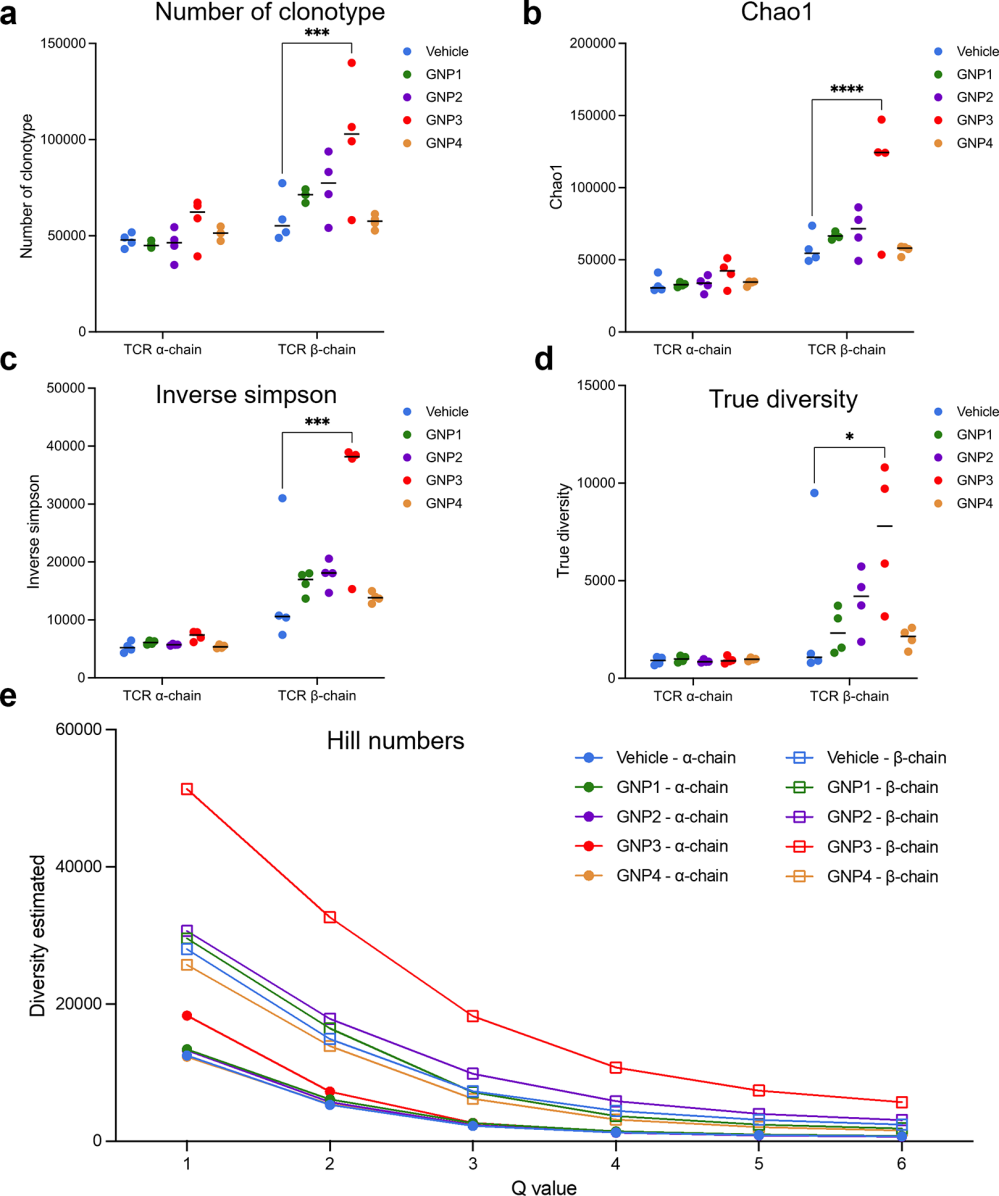
GNP3 increases TCR clonal diversity. (a–d) Number of clonotypes and clonal diversity estimated by Chao1, Inverse simpson, and True diversity. Each point represents an individual animal (*n* = 4). Lines represent the median. *p*-values calculated by two-way ANOVA. *p < 0.05; **p < 0.01; ***p < 0.005; ****p < 0.001. (e) Diversity (Hill numbers) profile. Data are presented as the mean of each treatment.

Clonal diversity has long been seen as another signal of significant and novel recognition events, either in response to pathogens or in the types of self-antigen recognition found in autoimmune diseases. It is therefore significant that we observed a very significant change in (CDR3 linked) clonality in the GNP3-shape regime, with a nearly 100% increase of unique CDR3 sequences in the TCR-β-chain (Figure 4a) and a dramatic increase in TCR-β-chain diversity (Figure 4b–e). Interestingly, we already see the onset of this change in the GNP2 regime, with the sharpest increase at GNP3, while the highly nonspherical GNP4 regime has unchanged clonal diversity for both TCR-α- and TCR-β-chains. This illustrates the point that the biological response to shape can be quite sharply defined and could perhaps be seen as an immunological “hot spot”. This finding is also consistent with previous observations^6^ that the GNP3-shape regime strongly regulates immune-related dendritic cell pathways that lead to elevated autoantibodies. Taken together, these observations suggest an intriguing link to shape-induced particle “hot spots” in autoimmunity.

The idea that biological recognition on the scale of some tens of nanometers involves distinctive mechanisms and induces novel downstream actions (not observed at the molecular scale) is potentially highly significant. Given the potentially wide-ranging implications, we wish to interpret our present observations conservatively, and in particular, we do not intend to stipulate a mechanism yet. In proposing a mechanism, it should also be noted that correlations between shape effects and biological outcomes are difficult to establish with certainty because other aspects of the particles (for example, volume^32^ and protein corona^1^) are also changing at the same time as shape. However, we believe that the unique patterns of cellular regulation that are being reported originate from a more specific recognition mechanism. Analogies to other discussions taking part in the scientific literature may be helpful as illustrations. In virology, it has long been known that the geometrical patterns of surface antigens affect the immunological outcome. These ideas are now pointing toward a mechanism in which distances between the virus surface ligands that prompt multiple simultaneous interactions between immune cell receptors, allowing for receptor clustering, are key.^33,34^ Indeed, recent reports suggest an interesting viral evasion strategy in which viral interligand distances grow to exceed the limits on target cell receptor oligomerization.^35^ It is worth noting that the intertip distances going from GNP3 to GNP4 are within the same ranges as in those discussions. Other biophysical surface studies also suggest that spatially correlated geometries may be a controlling factor in such observations. While these analogies are interesting, we do not yet have a sufficiently clear understanding of the whole range of immune related cell interactions to support such a mechanism. Clearly, this is a hypothesis that is worth investigating.

From a broader perspective, it appears to us that various threads in the literature are now converging toward the conclusion that nanoscale architecture is sensed and (to some degree) encoded into multiple tiers (including T cell) of biology. This has significant implications not just for practical issues, such as engineering of vaccine adjuvants, but more broadly in the way we see (and study) the nanoscale materials-biology interface in the future.

## Supporting Information Available

Figures including the microfluidic reactor for high reproducibility and narrow shape distribution for 5 nm seeds and GNPs, LPS detection of GNP1–4, T cell purity validation after enrichment, quality control of total RNA and cDNA libraries using the Bioanalyzer 2100; table including the hydrodynamic diameter and zeta potential of GNP1–4; details of the synthesis and characterization of GNPS, animal study design, quantification of GNPs in lymph nodes by ICP-MS, T cell enrichment, and TCR repertoire sequencing and analysis (PDF)
