# Supplementary material for: Integrated Methodology from Synthesis to in Vivo Study that Identifies Nanostructure Shape “Hot Spots” in T Cell Receptor Repertoire
Source: Nano Lett. 2025 Apr 21;25(17):7003–11. doi: 10.1021/acs.nanolett.5c00741 (PMC12046591; doi:10.1021/acs.nanolett.5c00741)
Supplement: Supplementary file 1 — nl5c00741_si_001.pdf [file nl5c00741_si_001.pdf]

Integrated Methodology from Synthesis to *in Vivo Study* that Identifies Nanostructure Shape "Hot Spots" in T Cell Receptor Repertoire

Yanqiu Ye[1,2,3,‡], Guohui Huang[1,2,‡], Wei Zhang[2,‡], Jiasheng Wu[1], Jianhao Wu[1], Yingxin Li[1], Xiaoxia Zhou[4], Jianbo Jia[4], Zengchun Xie[2], Bing Yan[4], Kenneth A. Dawson[1,2], Jingqi Chen[1,]*, Yi-Feng Wang[1,]* & Yan Yan[2,3,]**

1. Guangzhou Key Laboratory for Research and Development of Nano-Biomedical Technology for Diagnosis and Therapy, Guangdong Provincial Education Department Key Laboratory of Nano-Immunoregulation Tumor Microenvironment, Department of Oncology & Translational Medicine Center, The Second Affiliated Hospital & the Affiliated Cancer Hospital, Guangzhou Medical University, Guangzhou 510260 Guangdong, P.R. China.

2. Centre for BioNano Interactions, School of Chemistry, University College Dublin, Belfield, Dublin 4, Ireland.

3. School of Biomolecular and Biomedical Science, UCD Conway Institute of Biomolecular and Biomedical Research, University College Dublin, Belfield, Dublin 4, Ireland.

4. Institute of Environmental Research at Greater Bay Area, Key Laboratory for Water Quality and Conservation of the Pearl River Delta, Ministry of Education, Guangzhou University, Guangzhou 510006, P.R. China.

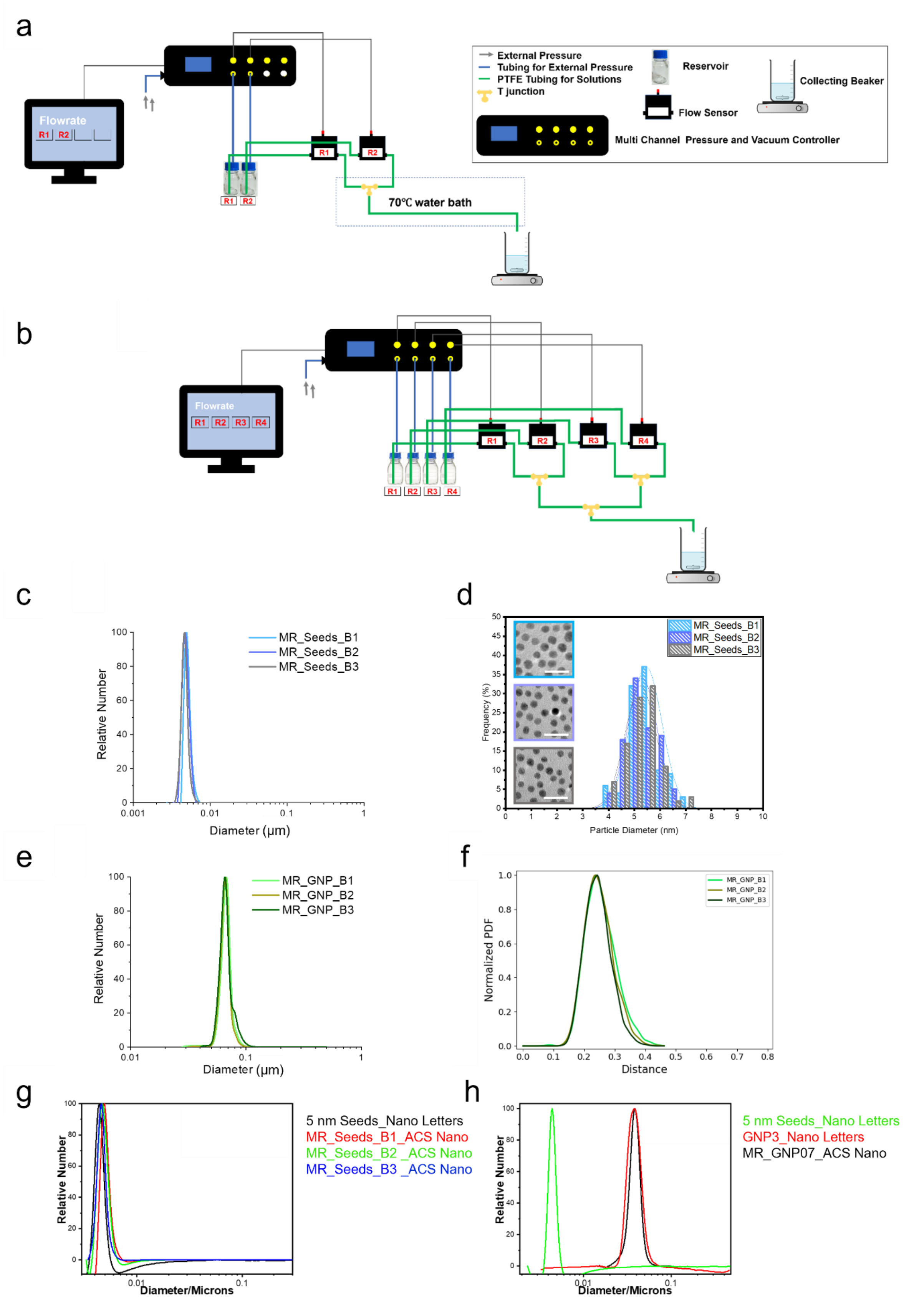

Figure S1. Microfluidic reactor (MR) for high reproducibility and narrow shape distribution for 5 nm seeds and GNPs. The reader should consult reference 6 for additional details of Figure

a-f. **(a)** Diagram of the microfluidic reactor synthesis setup for 5 nm seeds. **(b)** Diagram of the microfluidic reactor synthesis set up for microfluidic synthesis of GNP1-4 (MR_GNPs). Note that the small volume mixing and growth volume in the reactor suppresses particle-to-particle fluctuations and produces more uniform GNPs. Also, the small volume fast mixing makes seed growth highly efficient and exhausts seed reactants. Control of all flow, concentration and temperature conditions makes for high levels of reproducibility. (**c)** Differential Centrifugation Sedimentation (DCS) analysis has high precision for the size ranges of both seeds and GNPs and here shows the high reproducibility of different batches of 5 nm MR_Seeds. **(d)** Representative TEM micrographs and TEM size distribution. The scale bar is 20 nm. (**e)** example of DCS analysis illustrates the high reproducibility of different batches of GNPs (MR_GNP.) **(f)** Shape variance expressed as a probability distribution function (PDF) over distance showing the similarity of three batches of MR_GNP. The plot here may be understood as an independently calculated measure of shape derived from large numbers of electron micrographs of particles. **(g)** Reproducibility between different syntheses at different times and with different operators can be achieved. Such results require the synthesis to start with identical seeds, and here we show that seeds used for previous and present publications were the same. **(h)** Using identical conditions and the reproducible seeds completely independent synthesis can achieve GNP particle distributions without discernible differences. Here GNP3 particle produced in reference 6 are shown to be the same as GNP3 particles produced for this paper. DCS for seeds used in the present syntheses are overlaid to show the absence of residual seeds (or other particle side products). (Figure a-f) are reproduced from [Reference 1]. Copyright [2022] American Chemical Society.

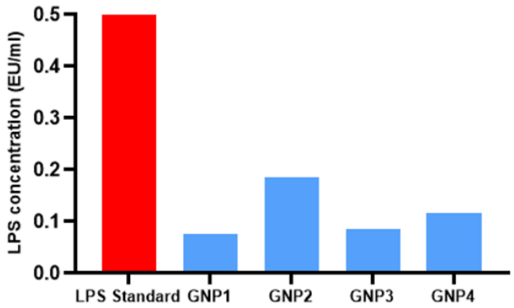

Figure S2. LPS detection of GNP1-GNP4.

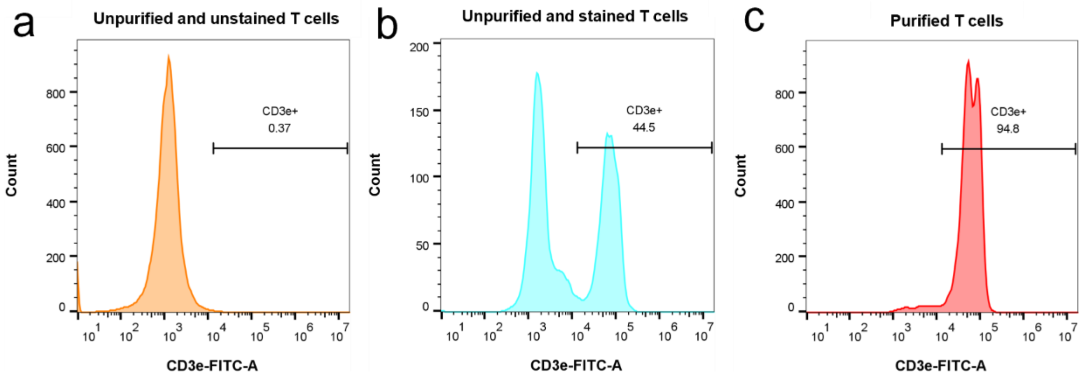

Figure S3. T cell purity validation after enrichment. (a) displays unstained T cells before enrichment, serving as a blank control. (b) and (c) illustrate T cell purity before and after enrichment.

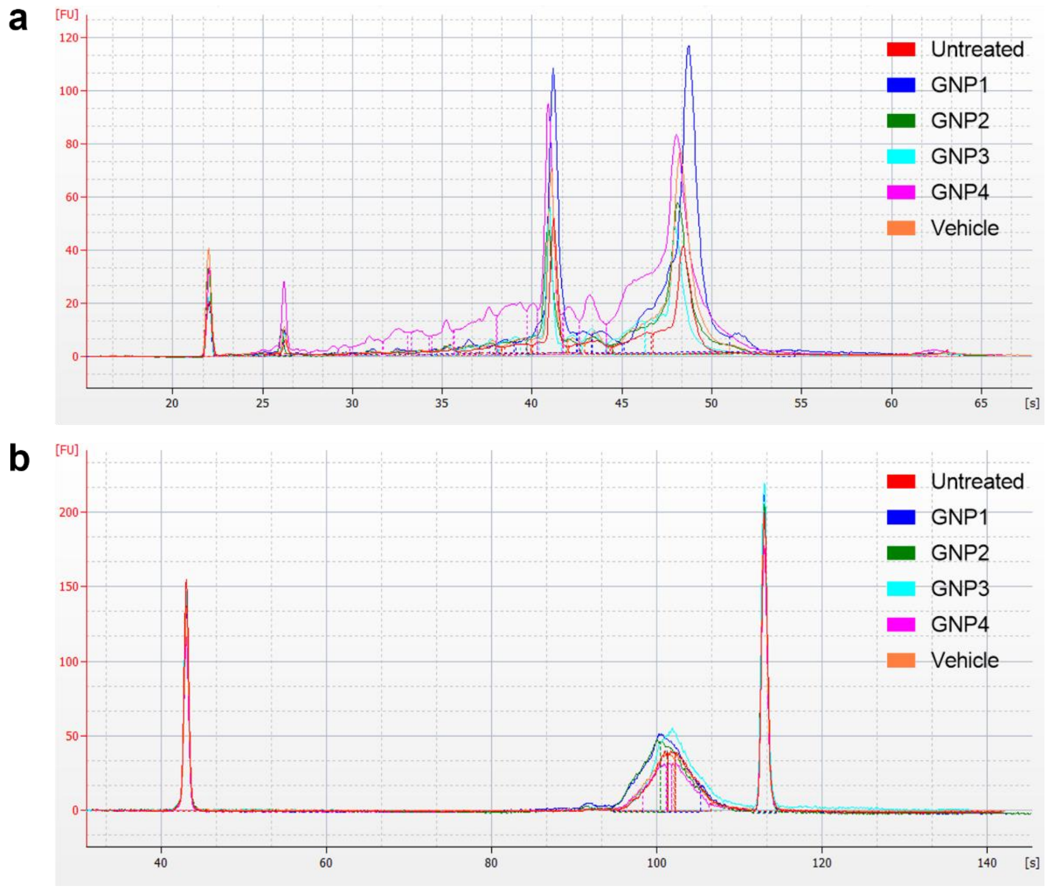

Figure S4. Quality control of total RNA and cDNA libraries using the Bioanalyzer 2100. (a) Overlay of the electropherogram for total RNA, detected with the RNA Pico 6000 chip. (b) Overlay of the electropherogram for the reverse transcribed cDNA libraries, detected with the DNA 1000 chip.

Table S1. Hydrodynamic diameter and Zeta potential of GNP1-GNP4.

	Hydrodynamic diameter (nm)	Polydispersity index (PDI)	Zeta potential in PBS (pH = 7.4) (mV)
GNP1	86	0.18	-28
GNP2	80	0.19	-24
GNP3	79	0.17	-25
GNP4	79	0.13	-23

EXPERIMENTAL PROCEDURES

Synthesis of GNPs. GNPs were synthesized using microfluidic systems as reported previously.[6] Firstly, we used a previously reported microfluidic set up to synthesize 5 nm gold nanoparticle seeds. The resulting seeds were sterilized with 0.2 μm filterer before proceeding to generate GNPs. Subsequently, we used another reported microfluidic set up to synthesize all the GNPs. The microfluidic synthesis was initiated by mixing gold precursor, seeds, and reducing solutions. The evolution of particle shapes was obtained by varying the ratio between the three solutions. Specific recipes are as follows. The flow rate is kept at 2000 μl/min, and reaction volume for each reservoir is 50 mL. The resulting particles were collected in a beaker with BSA (1 mg/mL, 10 mL) and stirred at 37 °C for 30 mins.

GNP1: Reservoir 1 ($HAuCl_4 \cdot 3H_2O$, 5×10^{-5} mol), Reservoir 2 (Seeds, 300 mL; Na_3Cit, 1.5×10^{-5} mol), Reservoir 3 (Hydroquinone, 3×10^{-5} mol), and Reservoir 4 (Hydroquinone, 3×10^{-5} mol).

GNP2: Reservoir 1 ($HAuCl_4 \cdot 3H_2O$, 5×10^{-5} mol), Reservoir 2 (Seeds, 300 mL; Na_3Cit, 1.5×10^{-5} mol), Reservoir 3 (Hydroquinone, 4×10^{-4} mol), and Reservoir 4 (Hydroquinone, 4×10^{-4} mol).

GNP3: Reservoir 1 ($HAuCl_4 \cdot 3H_2O$, 5×10^{-5} mol; $AgNO_3$, 5×10^{-8} mol), Reservoir 2 (Seeds, 300 mL; Na_3Cit, 1.5×10^{-5} mol), Reservoir 3 (Hydroquinone, 1×10^{-4} mol), and Reservoir 4 (Hydroquinone, 1×10^{-4} mol).

GNP4: Reservoir 1 ($HAuCl_4 \cdot 3H_2O$, 5×10^{-5} mol; $AgNO_3$, 1×10^{-7} mol; Glycerol, 500 mL), Reservoir 2 (Seeds, 300 mL; Na_3Cit, 1.5×10^{-5} mol), Reservoir 3 (Hydroquinone, 1×10^{-4} mol), and Reservoir 4 (Hydroquinone, 1×10^{-4} mol).

Characterization of GNPs. Particle shape was characterized by transmission electron microscopy (TEM) described previously.[6] The resulting TEM images containing well-spread GNPs were used to extract their contours for computational shape analysis following the protocol previously reported.[5] The number concentration of GNPs was measured by

nanoparticle tracking analysis (NTA).[35] The lipopolysaccharide (LPS) level of GNPs was evaluated by Limulus Amoebocyte Lysate (LAL) assay. The GNP dispersion with LPS level below 0.5 EU/mL was used for animal immunization.

Animal study design. All animal experimental protocols and analyses were conducted in accordance with the regulations and guidelines issued by the Chinese Government on administration of laboratory animals. The experimental procedure was approved by the Committee for Ethics on Animal Experiments of the Second Affiliated Hospital at Guangzhou Medical University. C57BL/6J mice were housed in cages, located in specific pathogen-free rooms with free access to water and food. The injected GNPs were prepared in GLP laboratory to avoid endotoxin contamination.

For the biodistribution study, 30 × C57BL/6J mice (15 males and 15 females) aged 6-8 weeks were randomly divided in 5 groups (n=6, 3 male and 3 female in each group): GNP1, GNP2, GNP3, GNP4, and Untreated. 1.5×10^{11} GNPs in 300 µl LPS-free water were administered subcutaneously into the loose skin over the interscapular area. Animals were sacrificed at different times after GNP injection: 1 day, 7 days, and 21 days. The mice were intraperitoneally anesthetized with 2% Avertin, exsanguinated by cardiac puncture, and then perfused with PBS to remove the blood from organs. The mice were then sacrificed by cervical dislocation. The superficial lymph nodes (including cervical LNs, axillary and brachial LNs, and inguinal and popliteal LNs) and other key organs (e.g., spleen, kidney, liver, heart, and lungs) were collected and stored at -80 °C before further processing. Several key steps were taken to minimize the delivery variations including multiple operators, and estimations and monitoring of dead volume of the syringe to ensure the accurate delivery volume.

For TCR repertoire study, 24 × C57BL/6J mice (12 males and 12 females) aged 6-8 weeks were randomly divided in 6 groups (n = 4, 2 male and 2 female in each group): GNP1, GNP2, GNP3, GNP4, Untreated, and BSA solution. 1.5×10^{10} GNPs in 300 µl BSA (1 mg/mL)

solution were administered subcutaneously into the loose skin over the interscapular area at day 0, day 21 and day 42. Animals were sacrificed as described above at day 84 after GNP injection, and LNs and key organs were collected. The excised axillary and brachial LNs were immediately processed in pre-cold cell media on ice to obtain single cell suspension.

Quantification of GNPs in lymph nodes by ICP-MS. Quantification of Au was carried out using an Agilent 7900 ICP-MS according to previously published protocol.[35] The collected organs were freeze-dried and accurately weighed in 15 mL polypropylene Corning tubes. To digest LNs and spleens, individual samples were treated with 1 mL aqua regia (MS grade) for 1 hour in a fume hood. To digest other larger organs, individual samples were treated with 3 mL of reverse aqua regia (MS grade) for 30 min in a fume hood, then supplemented with additional 3 mL of reverse aqua regia for further 30 min. Afterwards, microwave oven was used to completely digest the samples at 180 °C for 1 hour. The digested samples were heated at 100 °C for 3 hours by a metal bath heater to dispel the excess of acid via evaporation. The actual ICP-MS measurement was carried out on diluted samples with determined dilution factors. Based on the volume of digestion solution, gold concentration in the digestion solution, and dilution factors, we calculated the total mass of gold in each organ. We also measured the total mass of gold in the injected dose. Based on the mass ratio, the results were expressed as nanoparticle number in LNs.

T cell enrichment. The collected lymph nodes were gently ground with a syncing rubber piston on a 40 μm sterile filter (Falcon cat. no 352340) into a petri dish containing Hank's buffer (gibbon cat. no C14175500BT) (containing 3% heat inactivated FBS and 10 mM EDTA) on ice to obtain single cell suspension. T cells were isolated using MagniSortTM Mouse T cell Enrichment Kit (invitrogen cat. no 8804-6820-74) according to the manufacturer's protocol. Briefly, the single cell suspension was pelleted, resuspended in Hank's buffer, and incubated with MagniSortTM Negative Selection Beads. Then, magnet pulldown was carried out to

remove the unwanted cells. The supernatant was transferred to a fresh tube. Isolated T cells were pelleted and resuspended in PBS. The enrichment of T cells was confirmed by immunostaining with CD3e monoclonal antibody using flow cytometry (Agilent NovoCyte QuanteonTM). Briefly, 1×10^6 cells were first block with CD16/CD32 monoclonal antibody (eBioscience cat.no14-0161-81), and then stained FITC-conjugated CD3e monoclonal antibody (eBioscience cat.no 11-0031-81) at a concentration of 0.5 mg/mL for 40 min at 4 °C in the dark. FITC-conjugated Armenian Hamster IgG (eBioscience cat.no 11-4888-81) at a concentration of 0.5 mg/mL was used for isotype control. Cells were spun at 500 g for 5 min and washed with 1X PBS twice. Final pellet was resuspended in 200 µl 1X PBS for flow cytometry analysis.

Sample preparation and TCR repertoire sequencing. T cell total RNA isolation was performed using the RNeasy® plus micro kit (Qiagen cat. no 74034) following the manufacturer's instructions. RNA quality and concentration were checked immediately after the purification by Agilent 2100 bioanalyzer and Nanodrop. SMARTer Mouse TCR αβ Profiling Kit (Takara cat. no 634403) was used for synthesizing the first-strand cDNA and TCR repertoire library. Briefly, first-strand cDNA was synthesized as follows: 10 ng RNA, 1µl 10X Reaction Buffer, 2 µl TCR dT Primer, 4 µl 5X ultra Low First-Strand Buffer, 1 µl SMART-Seq v4 Oligonucleotide, 0.5 µl RNase Inhibitor, 2 µl SMARTScribe Reverse Transcriptase, and Nuclease-Free water to a total volume of 20 µl. PCR was performed as follows: 42 °C for 45 min, 70 °C for 10 min, 4 °C.

To amplify TCR α and β-chains, two rounds of PCRs were carried out. Specially, PCR1 was performed as follows: 20 µl cDNA reaction, 10 µl 5X PrimeSTAR GXL SP Buffer, 4 µl dNTP Mixture, 0.5 µl SMART Primer 1, 0.5 µl TCR α Mouse Primer 1 and TCR β Mouse Primer 1 mixture, 1 µl PrimeSTAR GXL SP DNA Polymerase, 14 µl Nuclease-Free Water. 95°C for 1 min, then run 21 cycles of 98 °C for 10 sec, 60 °C for 15 sec, 68 °C for 45 sec, and then 4 °C.

PCR2 was performed as follows: 1 μl PCR1 reaction, 10 μl 5X PrimeSTAR GXL SP Buffer, 4 μl dNTP Mixture, 0.5 μl TCR Primer 2 Forward HT Index primer, 0.5 μl TCR Primer 2 Reverse HT Index mixture, 1 μl PrimeSTAR GXL SP DNA Polymerase, 33 μl Nuclease-Free Water. 95 °C for 1 min, then run 18 or 21 cycles: 98 °C for 10 sec, 60 °C for 15 sec, 68 °C for 45 sec, and then 4 °C. Final PCR products were cleaned up by AMPure XP PCR purification Kit (Agencourt cat. no A63880) using a ratio of beads of 0.5X of the total PCR2 product volume. The cleaned final PCR product was eluted in 17 μl of RNasefree water. Quailty and concentration were checked by Agilent 2100 bioanalyzer prior to sequencing (BGI, Hiseq2500, 2X300bp).

TCR repertoire analysis. We used two main bioinformatic tools MiXCR and TRUST4 to analyze the V(D)J gene usage, CDRs, and clonal diversity. MiXCR (V3.0.13) tool (https://github.com/milaboratory/mixcr) based on java environment was used for alignment of reads for reporting the V(D)J germline segments. The resulting MiXCR alignment files were further analyzed by immunarch R package (V0.7.0) based on R language (V4.2.1) to get information of Clonetype number ("repExplore") and Clone diversity ("repDiversity"). TRUST4 (V1.0.8-beta) (https://github.com/liulab-dfci/TRUST4) based on Linux system was used to analyze V(D)J usage. In addition, we focused on using TRUST4 to reconstruct complementary-determining regions (CDR1, CDR2, and CDR3). GraphPad Prism 9 was used to plot the amino acid length of CDRs based on the TRUST4 output files.

REFERENCE

(1) Zhang, W.; Lopez, H.; Boselli, L.; Bigini, P.; Perez-Potti, A.; Xie, Z.; Castagnola, V.; Cai, Q.; Silveira, C. P.; de Araujo, J. M.; et al. A Nanoscale Shape-Discovery Framework Supporting Systematic Investigations of Shape-Dependent Biological Effects and Immunomodulation. *ACS Nano* **2022**, *16* (1), 1547-1559.
